# Research progress on biomarkers of immunoglobulin a vasculitis nephritis: from classical molecules to multi-omics system analysis

**DOI:** 10.3389/fimmu.2026.1794528

**Published:** 2026-04-29

**Authors:** Shuang Xu, Yan Xu, Yuefeng Bi, Ying Ding, Xia Zhang, Jian Zhang, Leying Xi, Xianqing Ren

**Affiliations:** 1 Pediatric Hospital of the First Affiliated Hospital of Henan University of Chinese Medicine, Zhengzhou, Henan, China; 2 School of Pediatrics, Henan University of Chinese Medicine, Zhengzhou, Henan, China; 3 The First Affiliated Hospital of Henan University of Chinese Medicine, Zhengzhou, Henan, China; 4 School of Pharmacy, Zhengzhou University, Zhengzhou, Henan, China

**Keywords:** biomarkers, immunoglobulin A vasculitis nephritis, omics, pathogenesis, precision medicine

## Abstract

Immunoglobulin A vasculitis nephritis (IgAVN) is the most common secondary glomerular disease in children, and the severity of renal involvement is a critical determinant of long-term prognosis. Although renal biopsy remains the gold standard for pathological diagnosis, its invasive nature and delayed indication limit its utility for early monitoring. With the advancement of precision medicine, identifying non-invasive and sensitive biomarkers has become an urgent clinical need. In recent years, beyond classical immune-inflammatory indicators, the application of high-throughput technologies such as genomics, proteomics, and metabolomics has provided a new dimension for the systematic characterization of the IgAVN molecular landscape. This review summarizes the current status of research on IgAVN biomarkers, focusing on the latest breakthroughs ranging from core immune molecules like Gd-IgA1 to multi-omics “fingerprints.” Furthermore, it critically analyzes the challenges currently faced in the clinical translation of these findings, aiming to provide a theoretical basis for establishing an early warning system and personalized diagnosis and treatment strategies for IgAVN.

## 1 Introduction

IgA vasculitis nephritis (Immunoglobulin A vasculitis nephritis, IgAVN), also known as Henoch-Schönlein purpura nephritis (HSPN), is not only the most serious complication of IgAV but also a leading cause of chronic renal failure in children (1). Clinical data indicate that approximately 30% to 50% of affected children develop renal involvement within 4 to 12 weeks after the onset of the rash (2). Although combined therapy with glucocorticoids and immunosuppressants is the current standard of care, a subset of patients responds poorly to treatment and faces the risk of progression to end-stage renal disease (ESRD) (3). Given the irreversibility of renal damage, identifying high-risk patients precisely in the early stages to initiate intervention is key to improving long-term outcomes.

However, the early diagnosis of IgAVN faces a dual dilemma of “lagged gold standard” and “risk of over-treatment.” Renal biopsy, as the gold standard for diagnosis, is not only invasive and difficult to repeat, but its evaluation system itself is also controversial. The traditional ISKDC classification over-relies on the proportion of crescents while neglecting chronic lesions such as tubulointerstitial changes. In contrast, the Oxford classification (MEST-C), derived from IgA nephropathy, can provide more comprehensive prognostic information; however, its applicability in IgAVN and its association with treatment decisions still require validation (4). Meanwhile, traditional indicators such as proteinuria and serum creatinine often become abnormal only after substantial renal injury has occurred. Although numerous novel biomarkers have been discovered in recent years, most studies have focused on differencing IgAVN patients from healthy children, neglecting the clinical distinction between “isolated cutaneous” IgAV and “nephritis-type” patients. If biomarkers lack sufficient specificity, they may lead to false-positive warnings, exposing low-risk children to the side effects of steroids or immunosuppressants unnecessarily. Therefore, identifying non-invasive biomarkers that accurately reflect kidney-specific injury and possess clear added clinical value is the core challenge in moving from basic research to precision medicine (5).

**Figure 1:**
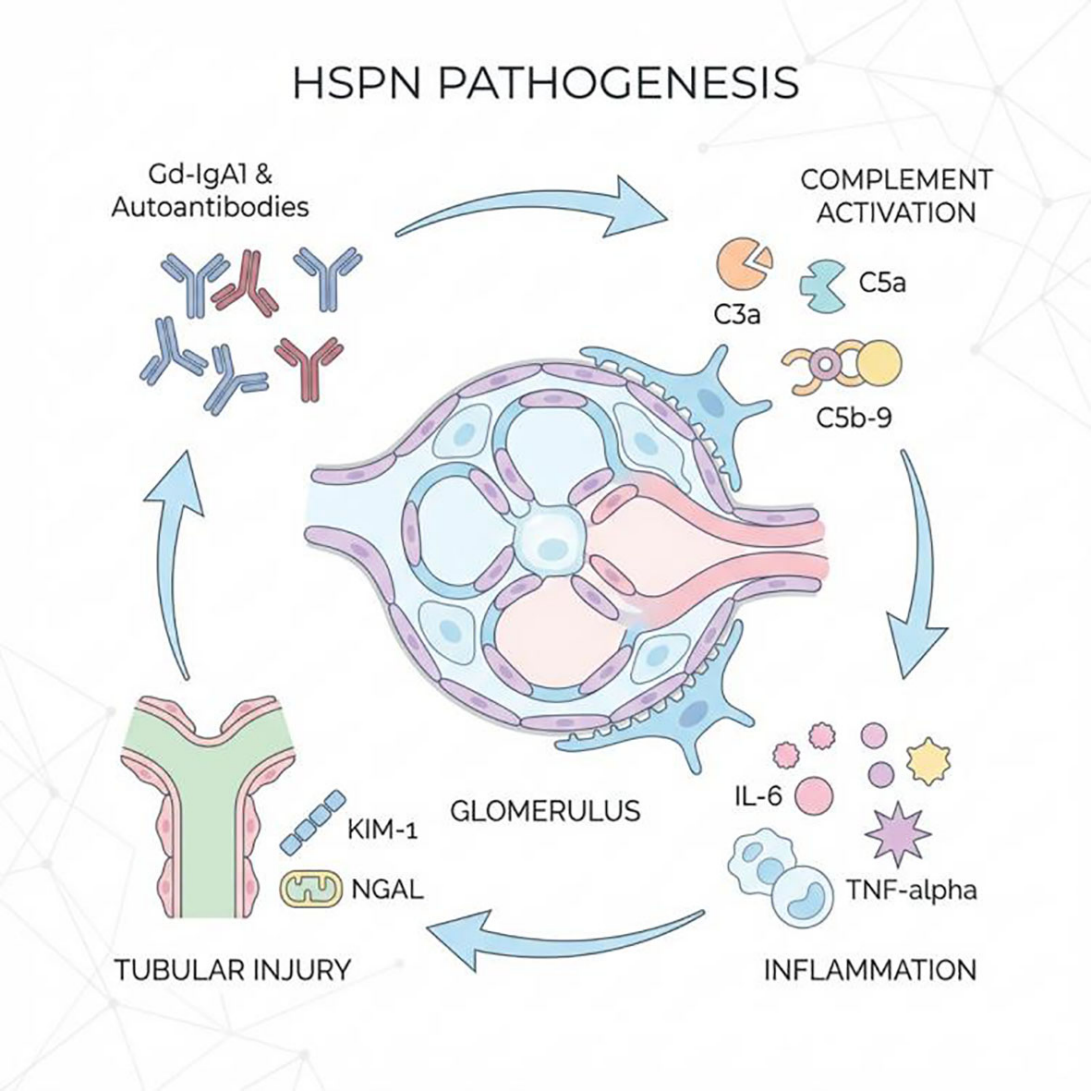
Schematic diagram of IgAVN biomarkers and pathogenic network This diagram illustrates the cascade process of IgAVN from genetic susceptibility (e.g., HLA variants) to immune abnormalities (Gd-IgA1 and immune complex formation), followed by local renal inflammation (complement activation, release of inflammatory cytokines) and tissue injury (release of renal tubular injury markers). It highlights the deposition of immune complexes within the glomerulus and the sources of major urinary markers (KIM-1, NGAL). The background integrates the potential molecular networks revealed by multi-omics (genomics, proteomics, metabolomics), reflecting the transition of biomarker research from single-molecule to systematic multi-dimensional analysis. Gd-IgA1, Galactose-deficient IgA1; C3a/C5a, Complement fragments; IL-6, Interleukin-6; TNF-α, Tumor necrosis factor-α; KIM-1, Kidney injury molecule-1; NGAL, Neutrophil gelatinase-associated lipocalin.

This article is a narrative review that synthesizes literature retrieved from major databases, including PubMed, Web of Science, Springer, China National Knowledge Infrastructure (CNKI), and Wanfang Data, up to January 2026, supplemented by manual screening of reference lists from key reviews and original studies. The search terms included “Henoch–Schönlein purpura nephritis”, “Immunoglobulin A vasculitis nephritis”, “IgAVN”, “HSPN”, “biomarker”, “omics”, “multi-omics”, “genomics”, “metabolomics”, “transcriptomics”, and “proteomics”. Studies were included if they focused on biomarkers or omics investigations in IgAVN/HSPN, and excluded if they were unrelated to the topic, had major design flaws, were of poor quality, or contained redundant content. No language restrictions were applied; however, for non-English articles, relevance was assessed based on the English abstract or full-text translation. Priority was given to studies offering mechanistic insights, clinical relevance, or translational potential. The aim of this review is to go beyond a simple listing of biomarkers. Following the logical framework of “pathogenic mechanism → clinical phenotype → systems mapping”, we will sequentially review core immune molecules based on pathogenesis, the expanded application of clinical biomarkers, the enhanced efficacy of multi-indicator combination strategies, and the dimensional leap brought by multi-omics technologies (**Figure 1**). Finally, we will discuss the challenges and future directions of clinical translation, with the goal of providing a theoretical basis for the precise diagnosis and treatment of IgAVN.

## 2 Research on pathogenesis-based candidate biomarkers

The pathogenesis of IgAVN involves multiple aspects such as immune abnormalities, complement activation, and inflammatory cascades. The molecular products of these pathological processes constitute the earliest sources of candidate biomarkers. Among them, the “four-hit” theory centered on galactose-deficient IgA1 (Gd-IgA1) establishes the theoretical cornerstone for the pathogenesis of IgAVN, and the series of molecules discovered around this theory have become the most disease-specific group of biomarkers.

### 2.1 Gd-IgA1 and its antibodies

Multiple studies have confirmed that IgAVN and IgA nephropathy (IgAN) share high similarity in renal immunopathological changes, both characterized by glomerular Gd-IgA1 deposition and elevated serum Gd-IgA1 levels (6–8). Serological evidence indicates that serum Gd-IgA1 levels are significantly elevated in patients with IgAV, particularly in those with renal involvement (IgAVN) (9). Notably, although Gd-IgA1 levels may be similar between IgAVN and IgAN (8), the elevation of Gd-IgA1 is considered a key threshold event for the progression of IgAV to IgAVN (10, 11). Pathological evidence also supports this view. For example, in cases of streptococcal infection-associated IgAVN, Gd-IgA1 deposition has been detected in both glomeruli and small skin vessels, indicating that vasculitis in the skin and kidneys shares the same molecular mechanism (12). Therefore, Gd-IgA1 is not only a critical pathogenic link in IgAVN but also serves as a core molecule forming the foundation for subsequent “multiple hits.”

However, Gd-IgA1 levels alone as a single biomarker have limitations, as their pathogenic effects largely depend on autoantibodies (mainly IgG) targeting Gd-IgA1 and the immune complexes they form (13). These pathogenic circulating immune complexes containing Gd-IgA1 deposit in the glomerular mesangial area, activate complement, and trigger inflammation, ultimately leading to renal injury. This represents the core step of the “four-hit” hypothesis in the pathogenesis of IgAVN (13, 14). Suzuki et al. (9) found that serum levels of Gd-IgA1 and its specific IgG autoantibodies were significantly elevated in IgAVN patients, with the latter positively correlating with proteinuria. The dual inhibitor of BAFF/APRIL, Telitacicept, when used to treat refractory pediatric IgAVN, not only effectively reduced proteinuria but also led to a significant decrease in circulating levels of Gd-IgA1 and IgA-immune complexes (such as IgG-IgA complexes). This therapeutic response confirms the central role of these immune complexes in driving renal inflammation (15, 16). Additionally, recent studies have shown that elevated plasma levels of soluble CD89-IgA complexes (CD89, also known as FcαRI, is a specific receptor for IgA) have an area under the curve (AUC) of 0.861 for early prediction of IgAVN, demonstrating better predictive performance compared to using Gd-IgA1 alone (17). Another study found that urinary IgA concentrations were significantly higher in children with IgAVN than in IgAV children without nephritis and healthy controls, suggesting its potential as a non-invasive biomarker for assessing nephritis (18).

Despite promising prospects, translating the detection of Gd-IgA1 and its antibodies into routine clinical practice still faces challenges, with the primary limitation being the lack of standardization in detection methods. Current methods for measuring serum Gd-IgA1 include lectin-based (e.g., VVA) enzyme-linked immunosorbent assay (ELISA) (19) and immunohistochemical (IHC) or immunofluorescence staining (IF) using specific monoclonal antibodies (e.g., KM55) (20, 21). However, the specificity of the KM55 antibody for detecting Gd-IgA1 is controversial. Studies have shown that it may be positive not only in IgAN and IgAVN but also in other immune complex-mediated glomerular diseases with IgA deposition (21), limiting its reliability as a specific diagnostic tool. Therefore, future efforts are needed to develop more reliable, reproducible, and standardized detection methods. Large-scale prospective studies are also required to validate the precise value of Gd-IgA1 and its antibodies (or related immune complexes) in risk stratification, monitoring disease activity, and predicting long-term renal outcomes, ultimately enabling their clinical translation (22).

### 2.2 Complement system activation products

Abnormal activation of the complement system plays a crucial role in the pathogenesis of IgAVN. Compared to serum markers, which may be influenced by systemic inflammation, complement components in urine can more directly reflect the local renal inflammation and injury status (23). Wright et al. (24) reported that the concentration of complement proteins (C3, C4, C5, C5a) in the urine of IgAVN children was significantly increased, and the combined detection of multiple complement proteins further improved diagnostic accuracy (AUC = 0.92). Marro et al. (25) found increased excretion of complement factor D, factor B, and mannose-binding lectin-associated serine protease 1 in urine, reflecting the activation of the alternative and lectin pathways. Furthermore, the urinary C4d/creatinine ratio has been confirmed to be independently associated with the severity of renal injury and poor prognosis in IgAVN (26). As novel therapies targeting complement pathways (e.g., factor B inhibitors) enter clinical trials, urinary complement markers are expected to be used for screening patients who may benefit from these targeted treatments and for monitoring their efficacy, thereby enabling individualized and precise management of IgAVN (27, 28).

## 3 From traditional indicators to novel effector molecules: expansion and application of clinical biomarkers

Although pathogenesis-based biomarkers (such as Gd-IgA1 and its antibodies, complement activation products) have made significant progress in revealing the nature of IgAVN, their clinical application is still limited by factors such as insufficient standardization of detection and cost-effectiveness. In clinical practice, the assessment of renal involvement and severity in IgAVN has long relied on a series of classic clinical indicators—the former reveals the “cause” of the disease, while the latter reflects the “effect” of injury, complementing each other. Therefore, clarifying the application value and limitations of clinical markers is a necessary prerequisite for understanding the current status of IgAVN diagnosis and treatment and finding a foothold for the clinical translation of novel markers.

### 3.1 Urine-related biomarkers

Clinically accepted urine biomarkers for IgAVN include 24-hour urinary protein, urinary protein/creatinine ratio, and urinary protein concentration (29). Additionally, urinary microalbumin performs excellently in predicting the presence of IgAVN, with AUC of 0.81-0.98 (30). Beyond these classic indicators, a series of novel urinary biomarkers show potential in early detection of renal tubular interstitial injury and predicting disease progression.

N-acetyl-β-D-glucosaminidase (NAG), as a sensitive indicator of proximal renal tubular injury, has been confirmed to distinguish IgAVN patients from IgAV patients without nephritis (30). Türe et al. (31) found that during the latent period before the appearance of traditional nephritis symptoms, urine kidney injury molecule-1 (KIM-1) and neutrophil gelatinase-associated lipocalin (NGAL) levels were significantly elevated in children with IgAV, indicating their value as ultra-early warning markers for predicting renal involvement. Zhang et al. (32) further confirmed that urinary KIM-1 not only reflects early renal injury but also dynamically monitors treatment response. The application of these markers is expected to overcome the lag of traditional proteinuria and hematuria detection, promoting early detection of occult renal injury in IgAVN.

Regarding changes in the renal internal environment, Mao et al. (33) confirmed that elevated urinary angiotensinogen (AGT) levels indicate intrarenal renin-angiotensin system activation and parallel the severity of proteinuria. The prospective study by Pillebout et al. (34) confirmed that urinary levels of IgA, IgG, IgM, IL-6, IL-8, IL-10, IgA-IgG complexes, and IgA-sCD89 complexes were significantly elevated in children with IgAVN compared to IgAV patients without nephritis, with urinary IgA and IgM demonstrating the highest diagnostic efficacy (AUC of 0.86 and 0.87, respectively). Berthelot et al. (35) further identified urinary IgA as a non-invasive biomarker with independent prognostic value in an adult cohort. The evolution from classic to novel biomarkers has greatly enriched the urinary biomarker spectrum for IgAVN.

### 3.2 Blood-related biomarkers

In addition to Gd-IgA1, the core pathogenic molecule, the discovery of various novel blood biomarkers reflecting different pathological processes provides a multi-dimensional perspective for the non-invasive assessment of renal damage in IgAVN.

Regarding the reflection of the severity of renal damage, for instance, serum tumor necrosis factor-α (TNF-α) levels are significantly correlated with renal function (eGFR) and renal pathological grading (ISKD classification) in adult IgAVN patients. Patients in class IV (the most severe) have significantly higher TNF-α levels than those in classes II and III (36). Zhang et al. (37) established serum α-smooth muscle actin (α-SMA) and hepatocyte growth factor receptor (c-Met) as non-invasive assessment indicators for the severity of IgAVN, and there was no significant difference in serum alpha SMA and c-Met levels between adult and pediatric patients. Wu et al. (38) found that serum apolipoprotein M (apoM) levels were higher in IgAVN patients with class I and II than in those with class III and IV, indicating that its level is negatively correlated with the ISKDC grading score in IgAVN patients. Low levels of apoM are an independent risk factor for predicting the occurrence of IgAVN.

In terms of early diagnosis and differential diagnosis, for example, the AUC of pentraxin 3 (PTX3) for diagnosing IgAVN reached 0.837, significantly better than CRP, and it is positively correlated with urinary microalbumin levels, making it an early sensitive marker for predicting the occurrence of IgAVN (39). Su et al. (40) found that serum levels of Midkine (MK) were significantly elevated in children with IgAVN, with a critical value of 295.58 pg/ml. The sensitivity and specificity of diagnosing IgAVN were 80.6% and 88.3%, respectively (AUC = 0.902), demonstrating extremely high diagnostic efficiency.

Furthermore, some studies have revealed new pathogenic mechanisms or monitoring pathways. The latest research found that high mobility group box 1 (HMGB1) is significantly elevated in IgAVN patients and is expected to become a potential tool for monitoring nephritis in IgAV patients. It may be directly involved in the pathogenic process through interactions with the RAGE receptor and Gd-IgA1 (41). Increased serum erythrocyte glutathione S-transferase (e-GST) activity can sensitively reflect impaired renal function, particularly suitable for monitoring children with borderline urine test results (42).

Whether it is a single biomarker in urine or blood, it is difficult to comprehensively capture the complex pathological process of IgAVN. Gd-IgA1 reflects immune initiation, complement products indicate inflammatory activation, KIM-1, NGAL, etc., indicate renal tubular damage, and TNF-α, MK, etc., are associated with disease severity—these biomarkers anchor different nodes in the pathogenic network. This inevitably leads to the consideration: can the combined detection of these complementary indicators achieve a “1 + 1>2” improvement in diagnostic efficacy?

## 4 Combined multi-marker detection significantly enhances IgAVN diagnostic efficacy

Given the limitations of single markers, constructing multi-marker combined detection models is an inevitable trend for improving diagnostic efficacy. A recent systematic review study found that urinary KIM-1(AUC 0.93), MCP-1(AUC 0.83), NAG(AUC 0.76-0.96) and AGT constituted the most promising biomarker combination for predicting IgAVN (30). Additionally, Li et al. (43) innovatively proposed combining the intestinal permeability marker Zonulin with the core immune indicator Gd-IgA1, enabling non-invasive precise diagnosis of IgAVN through serological testing. Research by Chinese investigators has confirmed: Ma et al. (44) found that the combined analysis of urinary AGT/Ucr, fibroblast-specific protein 1, and thrombin levels significantly elevated in children with IgAVN, with the first two being valuable for indicating renal injury; a logistic regression model established by Tian et al. (45) showed that the combined detection of urinary IgG, microalbumin, and transferrin could achieve a diagnostic specificity of 96.2%. This multi-dimensional, multi-target combined strategy significantly improves the sensitivity and specificity of early IgAVN diagnosis.

However, this combination is still limited to the “assortment-style” stacking of known molecules, making it difficult to discover entirely new Biomarkers or reveal unknown pathogenic pathways. To achieve a systematic depiction of the molecular panorama of IgAVN, it is essential to introduce higher-throughput research paradigms—omics technologies.

## 5 Omics technologies: a dimensional leap from single molecules to system maps

The introduction of omics technologies marks the entry of IgAVN biomarker research into a new era of systems biology. Unlike traditional methods investigating single molecules, omics technologies, through high-throughput data mining, comprehensively reconstruct the molecular network map of IgAVN from four dimensions: genetic susceptibility (genomics), transcriptional regulation (transcriptomics), protein execution (proteomics), and metabolic terminals (metabolomics) (46).

### 5.1 Genomics: mapping genetic susceptibility and ethnic specificity

Genomics research has revealed the complex genetic architecture of IgAVN, with variants in the HLA region recognized as core susceptibility factors. Multiple genome-wide association studies (GWAS) across different populations have confirmed that the HLA region is significantly associated with susceptibility to Henoch-Schönlein purpura (IgAV) and its nephritic phenotype (IgAVN). For example, studies in European, Han Chinese, and Finnish populations have consistently identified specific alleles of HLA class II genes (such as HLA-DQA1, HLA-DQB1, and HLA-DRB1) to be closely associated with disease susceptibility (47–49). A recent study on HLA gene polymorphisms in Croatian IgAV/IgAVN patients further supports these findings (50), indicating that HLA genes represent a major genetic factor in the pathogenesis of IgAV/IgAVN, with HLA class II genes showing the strongest association. Notably, the allele HLA-DRB1*14:01P exhibited even higher significance in IgAVN patients who developed nephritis.

In addition, other susceptibility gene loci have been identified in studies. For example, genetic variants of complement factor H have been found to be associated with IgAVN and the complement activation phenotype in IgAN (51). Another study suggested that the rs9428555 allele is associated with the onset of IgAV in Korean children (52). Batnozic et al. (53) investigated the association between HMGB1 and receptor for advanced glycation end products (RAGE) polymorphisms and the clinical course of different phenotypes in a pediatric IgAV population. The study found that HMGB1 rs1412125 is specifically associated with the occurrence of IgAVN. These findings suggest that risk stratification based on genetic background may become a source indicator for predicting IgAVN occurrence in the future.

However, current research is limited by sample size and ethnic differences, with insufficient statistical power for the discovered loci and a lack of functional validation. Therefore, future studies urgently need to conduct large-scale, multi-center, cross-ethnic validation and integrate functional experiments to clarify the exact role of these genetic variants in the pathogenesis of IgAVN.

### 5.2 Transcriptomics and single-cell technologies: deconstructing cell heterogeneity and regulatory networks

Transcriptomic studies have expanded the perspective to non-coding RNA regulatory networks and heterogeneity at the single-cell level. Bajželj et al. (54) combined RNA sequencing and machine learning to analyze skin tissues from IgAV patients, identifying potential biomarkers for renal injury in adult IgAV, highlighting molecules such as lipopolysaccharide-binding protein. Another study detected multiple differentially expressed lncRNAs in the peripheral blood of children with IgAVN, including ENST00000378432; PCR validation confirmed their association with the p53 signaling pathway and apoptosis-related genes, suggesting their involvement in disease pathogenesis (55). Focusing on immune- and apoptosis-related lncRNAs in IgAVN, Huang et al. (56) constructed an lncRNA-miRNA-mRNA regulatory network based on the competitive endogenous RNA (ceRNA) mechanism. Their study revealed 11 dysregulated lncRNAs, and further clinical validation indicated that specific lncRNAs within this network may serve as potential prognostic biomarkers for pediatric IgAVN patients. Levin et al. (57) performed RNA sequencing on glomeruli and tubulointerstitium from 71 adult and 13 pediatric IgAN/IgAVN patients, as well as 11 living donor kidneys, systematically revealing fundamental differences in transcriptional profiles between pediatric and adult patients for the first time. The results showed that pediatric glomeruli were enriched in endoplasmic reticulum and mitochondrial metabolic pathways, while tubulointerstitium was enriched in T-cell activation pathways, confirming age-dependent differences in pathological mechanisms at the molecular level.

More distinctively, single-cell RNA sequencing (scRNA-seq) technology enables “high-resolution” analysis of intrinsic kidney cells and peripheral blood immune cells. Recent studies have shown that Ye et al. (58) conducted scRNA-seq analysis on renal tissues from healthy control children and children with IgAN and IgAVN, finding differences in hypoxia-inducible factor 1α gene expression changes and energy metabolism between the two diseases, providing valuable insights into their association. Zhou et al. (59) analyzed peripheral blood mononuclear cells from healthy donors and IgAV patients using scRNA-seq and multiparameter flow cytometry, revealing that cytotoxic effector T-cell subsets were enriched in cutaneous IgAV and renal IgAV, while plasma cells, B cells, and Tfh cells were enriched in articular and abdominal IgAV, reflecting the dynamic nature of immune responses throughout the progression of IgAV with different clinical manifestations.

Although the aforementioned tissue- and blood-based studies have preliminarily revealed the transcriptomic characteristics of IgAVN, the search for more clinically applicable non-invasive markers remains a current focus. Urine, as a non-invasive source of biological samples, has been confirmed to identify almost all types of kidney cells (60). Therefore, systematically applying scRNA-seq technology to urine cell analysis holds promise for providing novel, dynamically monitorable diagnostic markers for IgAVN, expanding its application prospects in the precision diagnosis and treatment of kidney diseases. Additionally, microRNA (miRNA) in urinary exosomes also shows great potential. For example, the expression level of hsa-miR-383-5p has been found to be significantly correlated with urinary protein quantification in children with IgAVN and may participate in disease progression by targeting pro-fibrotic and inflammatory pathways, representing another highly promising novel non-invasive biomarker (61).

### 5.3 Proteomics: deciphering non-invasive humoral markers and effector networks

Proteomics directly reflects the executors of biological functions and is the main battleground for discovering non-invasive blood and urine markers. In the field of serum proteomics, He et al. (62) utilized high-sensitivity nano-scale ultra-high performance liquid chromatography-mass spectrometry (nanoLC-MS/MS) technology to investigate changes in serum proteome structure among patients with IgAV (n=6), IgAVN (n=6), and healthy volunteers (n=7). Combined with enzyme-linked immunosorbent assay (ELISA) validation, the results indicated that AGT levels are associated with the progression of IgAVN. Liu et al. (63) employed magnetic bead separation combined with MALDI-TOF MS technology to perform proteomic analysis on serum samples from IgAV patients before and after treatment (n=38) and healthy volunteers (n=22). Combined with ELISA validation, the results revealed that serum complement C4A and IgA could serve as early independent risk factors for distinguishing IgAV from IgAVN.

In urine proteomics research, Fang et al. (64) used data-independent acquisition (DIA) technology based on LC-MS/MS to detect and analyze the urine proteome of 30 IgAVN patients and 29 healthy controls. Combined with ELISA validation, they found that tenascin may be a novel biomarker for the early diagnosis of renal injury in IgAVN. Huang Yanjie et al. (65) applied label-free quantitative proteomics technology to compare and analyze urine samples from 10 children with ISKDC pathological type II and 30 children with type III IgAVN. The results showed that the expression of annexin A5 (ANXA5) is closely related to renal crescent formation, suggesting it could serve as a candidate biomarker for predicting early crescent formation in IgAVN (sensitivity 80%, specificity 76.7%). Zhu et al. (66) further employed a parallel accumulation-serial fragmentation (PASEF) proteomics method combined with DIA to screen the urine proteome of 8 children with IgAVN and 8 healthy controls. Through ELISA validation, the results indicated that zinc-alpha2-glycoprotein 1 (AZGP1) is significantly elevated in the urine of children with IgAVN and could serve as a specific biomarker for early diagnosis of the disease.

In renal tissue proteomics, Gao et al. (67) used isobaric tags for relative and absolute quantitation (iTRAQ) technology to perform proteomic analysis on kidney tissues from children with IgAVN (n=3) and nephrectomy controls (n=3). Combined with validation using IHC and PCR techniques, the results suggested that CDC42 and CTNNB1 may be potential candidate biomarkers involved in the pathogenesis of IgAVN.

Blood proteomics offers high sensitivity and mature technology but is interfered with by high-abundance proteins (such as albumin and immunoglobulins), making low-abundance proteins related to IgAVN (such as TNF-α and IL-6) easily masked. In contrast, urine proteins have a relatively simple structure and good stability, but standardized processing procedures are lacking, and protein concentrations are low, resulting in clinical applications lagging behind serum proteomics. Although renal tissue proteomics can directly obtain pathological-specific protein expressions in the kidney glomerulus and renal tubules, it is limited by the small sample size of biopsies, difficulty in acquisition, and tissue heterogeneity, making dynamic monitoring and large-scale cohort validation challenging (68). Additionally, the validation of the above biomarkers mostly relies on techniques such as ELISA, IHC, and PCR. Future studies could further validate the efficacy of these biomarkers by combining targeted parallel reaction monitoring technology.

### 5.4 Metabolomics: capturing early metabolic perturbations of renal involvement

Located downstream in biological systems, metabolomics can sensitively capture minute metabolic perturbations prior to phenotypic changes. Sun et al. (69) conducted serum metabolomics analysis on children with IgAV (n=52), IgAVN (n=57), and healthy children (n=53) using ultra-performance liquid chromatography quadrupole time-of-flight tandem mass spectrometry (UPLC-Q-TOF-MS/MS). The results revealed that specific metabolites such as (S)-3-hydroxyisobutyric acid were associated with the progression of IgAVN. When combined with the clinical indicator D-dimer, the predictive sensitivity (94.7%) and specificity (80.8%) were high. Demir et al. (70) performed plasma metabolomics analysis on IgAV (n=39), IgAVN (n=6), and healthy controls (n=6) using Q-TOF LC/MS. The results indicated that various metabolites, including dihydroxyacetone phosphate, were elevated before the onset of renal involvement, suggesting their predictive value. Yu et al. (71) analyzed 30 cases each of IgAV, IgAVN, and healthy children through non-targeted urine metabolomics using LC-MS/MS. The results showed that propionylcarnitine and indoxyl sulfate might be potential biomarkers for predicting the occurrence of IgAVN. Additionally, a matched serum-urine metabolomics analysis of 90 IgAVN patients (46 with severe renal injury [IgAVN(+)] and 44 with minimal symptoms [IgAVN (–)]) revealed that choline and cis-vaccenic acid emerged as key discriminators of disease severity, and thus are proposed as potential predictive biomarkers for IgAVN progression (72). These changes in small molecules constitute the “metabolic fingerprint” of early IgAVN, providing the possibility for clinical intervention before irreversible damage occurs.

### 5.5 Multi-omics integration: constructing cross-dimensional interaction networks

Faced with the complexity of the disease, single omics approaches struggle to provide a complete picture. Cross-validation and deep integration of multi-omics data have become an inevitable trend. Deng (73) adopted a strategy combining proteomics (MRM technology, iTRAQ labeling quantitative technology, and SWATH label-free quantitative technology), metabolomics (GC-MS), and metallomics (ICP-TOF MS technology) to conduct systematic research on urine disease markers of IgAVN. The results showed that seven metabolites such as azelaic acid, copper, and vanadium may be potential markers indicating IgAV kidney injury, providing reference data and preliminary foundation for future extensive multi-omics studies of this disease. Xie et al. (74) used transcriptome sequencing and quantitative proteomics based on TMT to obtain serum transcriptomic and proteomic profiles of IgAVN patients with different pathological types. The results found that 58 mRNAs and 1 protein continuously changed during the development of IgAVN and could be used as potential markers at various stages of IgAVN progression. These studies demonstrate the unique advantages of multi-omics strategies in systematically analyzing the molecular network of IgAVN and discovering novel biomarkers, which is an important direction for achieving precise diagnosis and treatment in the future.

## 6 Summary and outlook

### 6.1 Diagnostic mode shift from single molecules to multi-dimensional omics networks

Research on IgAVN biomarkers has transitioned from early single-indicator detection to multi-dimensional, high-throughput omics network analysis. Early studies primarily relied on routine urine and specific protein monitoring (proteinuria quantification, renal function), which, while simple, lacked specificity. With advances in immunology and molecular biology, inflammatory mediators, complement products, and renal tubular injury markers can reflect renal involvement earlier. Compared to the lag of traditional indicators like proteinuria, omics markers (especially urinary exosomal miRNA or metabolic fingerprints) can capture subtle perturbations at the molecular level during the “subclinical measures phase” before substantial renal tissue remodeling occurs. This ultra-early warning capability is key to achieving early interception. In particular, the introduction of genomics, proteomics, and single-cell transcriptomics has not only revealed the complex molecular regulatory network of IgAVN but also discovered novel markers including HLA genetic variants (47) and specific protein fingerprints (AZGP1) (66). Future strategies based on multi-marker combined detection (immune indicators combined with metabolic fingerprints) will significantly enhance diagnostic sensitivity and specificity, driving IgAVN diagnosis from “single-point molecular breakthrough” to “systematic analysis.”

### 6.2 Clinical translation still faces bottlenecks of missing validation and insufficient standardization

Although potential biomarkers are emerging endlessly, very few can truly be applied to routine clinical testing, and their translational application faces severe challenges. First, insufficient external validation and significant age bias: most studies remain at the small-sample discovery stage, and the vast majority of biomarker evidence originates from pediatric cohorts. There is a severe lack of research across age groups, as well as a deficiency in large-scale, multi-center, prospective cohort external validation. This results in doubts about the robustness and generalizability of the markers, making it difficult to establish a widely accepted clinical consensus. Second, lagged mechanism elucidation: although high-throughput omics screen out numerous differential molecules (such as specific lncRNAs or metabolites), deep analysis of their biological functions and specific roles in IgAVN pathogenesis is still lacking, limiting their potential as therapeutic targets. Third, lack of standardized systems: differences in sample processing, detection platforms (e.g., mass spectrometry, ELISA, sequencing platforms), and data analysis standards across studies make horizontal comparison of results difficult, hindering data aggregation and integration. Finally, balance between non-invasiveness and cost: while renal biopsy is the gold standard but invasive, high-throughput omics testing involves high costs and technical thresholds. How to achieve low-cost, non-invasive, and precise monitoring in routine clinical testing and find “cost-effective” alternative indicators remains a practical problem urgently needing solution.

### 6.3 Precision typing and artificial intelligence will drive future individualized diagnosis and monitoring

In existing studies, only a few biomarkers [e.g., serum TNF-α (36), serum apoM (38)] have been reported to correlate with the ISKDC classification, and these associations are largely limited to overall grading, lacking systematic correlation analyses with specific pathological lesion types (e.g., cellular/fibrocellular crescents, glomerulosclerosis, tubulointerstitial inflammation/fibrosis). To date, studies employing fine-mapping using individual Oxford MEST-C components remain extremely scarce (e.g., elevated urinary C4d/Cr ratio (26)has been associated with severe Oxford C lesions), and no biomarker has been independently correlated with chronicity indicators such as the T score (tubular atrophy/interstitial fibrosis). These limitations severely undermine the clinical utility of biomarkers for the non-invasive assessment of pathological activity and chronicity. Therefore, future research on biomarkers for IgAVN should focus on precision subtyping and individualized diagnosis and treatment. The primary task is to establish a mapping relationship between biomarkers and specific pathological lesion types. Given the limitations of the ISKDC classification and the growing adoption of the Oxford classification in IgAVN, future biomarker studies should aim to: (1) Reflect active lesions: identify urinary or blood-derived molecules capable of specifically differentiating cellular/fibrocellular crescents (potentially reversible) from fibrous crescents/global sclerosis (irreversible), such as the recently reported association between urinary ANXA5 (65)and crescent formation, although further validation of its ability to discriminate crescent activity is needed; (2) Quantify chronic injury: develop noninvasive indicators associated with tubular atrophy/interstitial fibrosis (T score) to enable dynamic monitoring of chronicity progression; (3) Integrate pathological subtyping: clarify the specific pathological dimensions (e.g., high T score or crescent formation) to which novel biomarkers (e.g., AZGP1 (66) map in validation cohorts, thereby endowing noninvasive test results with clear pathological implications to guide precise diagnosis and treatment. On the other hand, it is essential to construct multimodal predictive models that integrate clinical phenotypes, pathological quantitative scores, imaging findings, and multi-omics data, using artificial intelligence (AI) and machine learning algorithms to build high-precision risk prediction models (5), thereby achieving a refined transition from determining the “presence or absence of nephritis” to identifying “which pathological changes are present” and assessing “the risk of progression.” Simultaneously, deepen single-cell and spatial omics research: utilize scRNA-seq and spatial transcriptomics technologies to map the fine landscape of the renal immune microenvironment, precisely locate pathogenic cell subsets (such as cytotoxic effector T cell subsets) ^[59]^, and analyze intercellular communication networks, providing a basis for developing cell-specific targeted drugs. Furthermore, promote translational medicine research: establish a standardized, high-quality biobank covering children and adults, conduct prospective multicenter clinical trials to validate the diagnostic and predictive value of potential biomarkers (such as Gd-IgA1, sCD89-IgA complex) (9, 17) in different age groups, and accelerate their translation into clinical testing kits. Focus on developing novel liquid biopsy technologies based on urinary exosomes and so on to realize real-time, non-invasive “visualization” monitoring of renal pathological states, ultimately establishing a molecular typing system for IgAVN to guide individualized precision treatment and improve the long-term prognosis of affected children.

In summary, from classic urinary protein quantification to core pathogenic molecules such as Gd-IgA1, and further to the systemic landscape revealed by multi-omics, research on biomarkers for IgAVN has evolved over several decades and is gradually approaching the ultimate goal of “non-invasively reflecting renal pathology.” In the future, with the standardization of detection techniques, the expansion of large-scale validation cohorts, and the deep empowerment of artificial intelligence, a biomarker-based molecular classification system for IgAVN is expected to transition from vision to reality, ultimately achieving the leap from “empirical treatment” to “precision medicine.”
